# *In silico* analysis of Glanzmann variants of *Calf-*1 domain of α_IIb_β_3_ integrin revealed dynamic allosteric effect

**DOI:** 10.1038/s41598-017-08408-w

**Published:** 2017-08-14

**Authors:** Matthieu Goguet, Tarun Jairaj Narwani, Rachel Petermann, Vincent Jallu, Alexandre G. de Brevern

**Affiliations:** 1 Platelet Department Unit, INTS, F-75739 Paris, France; 2 INSERM, U 1134, DSIMB, F-75739 Paris, France; 3 Univ Paris Diderot, Sorbonne Paris Cité, UMR_S 1134, F-75739 Paris, France; 4 Institut National de la Transfusion Sanguine (INTS), F-75739 Paris, France; 5 Laboratoire d’Excellence GR-Ex, F-75739 Paris, France

## Abstract

Integrin α_IIb_β_3_ mediates platelet aggregation and thrombus formation. In a rare hereditary bleeding disorder, Glanzmann thrombasthenia (GT), α_IIb_β_3_ expression / function are impaired. The impact of deleterious missense mutations on the complex structure remains unclear. Long independent molecular dynamics (MD) simulations were performed for 7 GT variants and reference structure of the *Calf-*1 domain of α_IIb_. Simulations were analysed using a structural alphabet to describe local protein conformations. Common and flexible regions as well as deformable zones were observed in all the structures. The most flexible region of *Calf-*1 (with highest B-factor) is rather a rigid region encompassed into two deformable zones. Each mutated structure barely showed any modifications at the mutation sites while distant conformational changes were observed. These unexpected results question the relationship between molecular dynamics and allostery; and the role of these long-range effects in the impaired α_IIb_β_3_ expression. This method is aimed at studying all α_IIb_β_3_ sub-domains and impact of missense mutations at local and global structural level.

## Introduction

**Figure 1:**
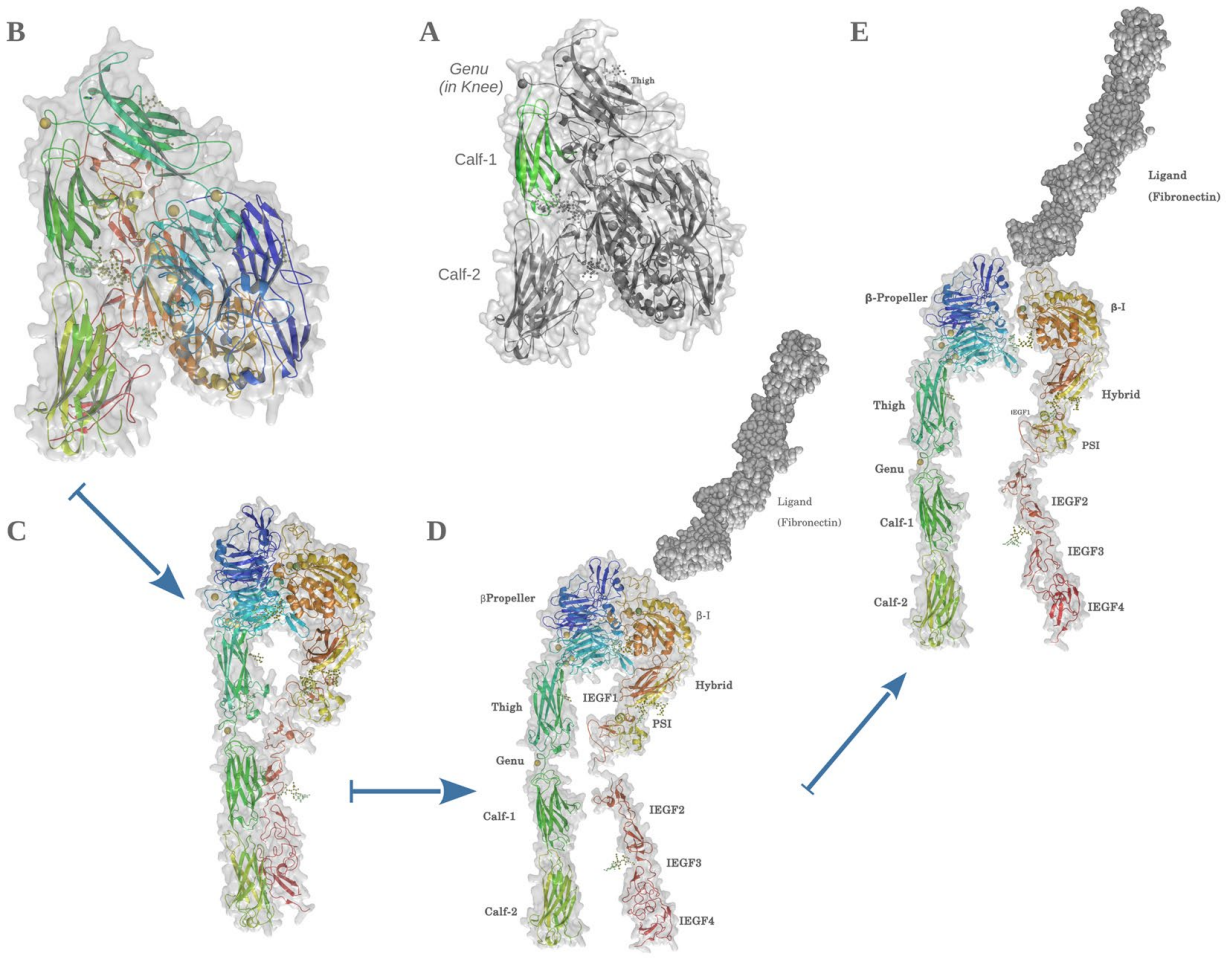
Closed to open transition of α_IIb_β_3_. (**A**) The closed form of α_IIb_β_3_ ectodomain, with *Calf-1* domain highlighted in green. Rest of the structure is depicted in dull grey to bring clarity. Structural organization of ectodomain is labelled. The rainbow schema of colours on secondary structures represents the α_IIb_β_3_ structure. Regions in green-blue spectra mark the α_IIb_ subunit and yellow-red spectra mark the β_3_ subunit. (**B**) Closed inactive form of α_IIb_β_3_. The structure is bent along the plane of knee domains. (**C)** Extended α_IIb_ headpiece with β_3_ leg resting alongside the α_IIb_ leg. (**D**) Extended β_3_ conformation: The β_3_ headpiece has intrinsic conformational changes at C-terminus leading to an outward pull of the β_3_ leg. (**E**) Extended α_IIb_β_3_ conformation: In the last stage, β_3_ headpiece pulls out creating a ligand-binding cavity between the two headpieces. Mg^++^ constituting MIDAS can be seen as a green sphere in the cavity, while the ligand Fibronectin (dull grey dots) approaches the glycoprotein. All metal ions are shown as solid spheres with golden representing Ca^2+^ while green representing Mg^2+^. Polysaccharides (N-acetyl glucosamines and Mannose) are shown in ball and stick representations. Please notice that the open forms had been modelled from the closed structure according to expected conformations using Modeller_v_9.16 and images are generated by PyMol_v_1.7.0.

In humans, the Integrins protein superfamily consists of 24 heterodimeric receptors resulting from different combination of 18 α and 8 β subunits. Integrins are highly dynamic glycoprotein (GP) involved in cell-cell or cell-matrix interactions^1^. Upon activation the integrin α_IIb_β_3_ binds plasmatic fibrinogen leading to platelet aggregation and thrombus formation (Primary haemostasis). Some well-known human integrin structures are: α_x_β_2_, α_v_β_3_ (cell-matrix adhesion) and α_IIb_β_3_ (cell-cell adhesion). α_v_ can associate to β_1,3,5,6,8_ subunits while α_IIb_, that is specific to platelets and megakaryocytes cells, only associates to β_3_^1^. The α_IIb_β_3_ structure is organized into 3 distinct regions; an N-terminus extracellular ectodomain, a single spanning transmembrane (TM) region and a C-terminus cytoplasmic region. The cytoplasmic region in α subunit is very swift (~20 residues) while in β subunit it extended up to 46 residues in length and constitutes an important node for signalling. Both α and β TM regions are single spanning, and consist of 22 residues each. The ectodomain is relatively huge with 959 and 693 residues in α and β subunits respectively. Figure 1 depicts transition steps from the inactive conformations of α_IIb_β_3_ (crystallized closed structure) to its theoretical open liganded active form. A complete structure of the open forms of the ectodomain with or without ligand remains to be crystallized. The ectodomain is further divided into four regions: headpiece, knee, legs and tails. The headpiece that carries the ligand-binding site consists of the *β-propeller* domain of α_IIb_ and the *β-I* domain of β_3_. The α_IIb_ *β-propeller* domain consists of a 7 bladed fold with four Ca^2+^ ions coordinated with β-hairpin loops connecting the antiparallel β-strands (see Fig. 1A). The *β-I* domain of β_3_ mainly consists of α helices and loops with coordinated metal ions Ca^2+^ and Mg^2+^ constituting a MIDAS (Metal Ion Dependent Adhesion Sites) with an ADMIDAS (Adjacent to MIDAS) and SyMBS (Synergistic Metal Binding Site). These sites play critical role in opening the α_IIb_β_3_ binding site and helps in ligand binding^2^. Following *β-propeller*, the α_IIb_ leg is composed of the *Thigh* domain, the *Genu* (*knee*), the rigid *Calf-*1 and *Calf-2* domains. The short loop of α_IIb_ Genu coordinates with a divalent calcium^3^. This metal ion might help in stabilizing *Calf-1* and *Thigh* domains interface during the opening of the structure following the activation process (angular shift at *Genu*). The α_IIb_ leg is rigid and provides a framework to the entire ectodomain. The β_3_ *β-I* domain of the headpiece is followed by the *Hybrid*, *PSI*, and 4 I*EGFs* (Integrin Epidermal Growth Factor) domains. A short knee joined the *IEGF-1* with *IEGF-2* domains. The β_3_ leg consists of *IEGF-2* to IEGF-4 whose C-terminus ends in the ankle domain (tail). α_IIb_ and β_3_ transmembrane and cytoplasmic domains are not shown. The activation state of α_IIb_β_3_ is tightly controlled by inside - out signalling resulting from platelet activation by multiple exogenous factors (physiological plasmatic agonists, exposed sub-endothelial matrix …). The α_IIb_ headpiece opens up creating an angular shift between *Thigh* and *Calf-1* domains (Fig. 1C) meanwhile, the β_3_ leg and tail remains parallel to the α_IIb_ leg. Thereafter, opening of the β_3_ headpiece pulls the β_3_ legs outward resulting into an extended open conformation (Fig. 1D,E) that can bind plasmatic fibrinogen at the MIDAS, which is constituted by elements of both the headpieces^4, 5^. Finally, fibrinogen binding leads to outside-in signalling.

The human integrin α_IIb_β_3_, a platelet surface fibrinogen receptor, is responsible for platelets aggregation, a key process in primary haemostasis and thrombus formation^6^. Defective primary haemostasis leads to two severe life-threatening bleeding disorders: (1) Glanzmann thrombasthenia (GT), a rare autosomal recessive genetic disease associated with impaired α_IIb_β_3_ expression and/or function^7^; and (2) Fetal/neonatal alloimmune thrombocytopenia (FNAIT) results from fetal/neonatal platelet destruction by maternal antibodies in mothers lacking an alloantigen inherited from the father. FNAIT clinical consequences range from no symptoms to intracranial haemorrhages with a risk of neurological sequel and/or fetal/neonatal death^8^.

Both diseases result from α_IIb_ and β_3_ gene polymorphisms. In GT, more than 300 mutations have been identified in α_IIb_ or β_3_ genes. Most of them are reported in https://sinaicentral.mssm.edu/intranet/research/glanzmann. In FNAIT, neither the expression nor function of α_IIb_β_3_ is affected but missense mutations results in amino acid (aa) substitutions that define Human Platelet Antigens (HPA). All human platelet alloantigens are described in the HPA database (http://www.ebi.ac.uk/ipd/hpa/). The effects of these amino acid substitutions on α_IIb_β_3_ structure remain largely unknown. We are interested in understanding how amino acid substitutions in GT can impact the *Calf*-*1* domain of α_IIb_β_3_ structure and its structural dynamics.

Very few structures of α_IIb_β_3_ have been crystallized and only one contains the whole α_IIb_β_3_ ectodomain (PDB id 3FCS^9^) in closed conformation (Fig. 1B). Using this structure, we showed that the β3 Lys253Met GT mutation impaired key ionic interactions between the α_IIb_ *β-propeller* and the β_3_ *β*-*I* like domain^10^. However static models cannot depict all mutation-induced effects on a highly dynamic structure.

In our previous studies, molecular dynamics (MD) simulations were used to compare the structures of the β3 L33 and P33 forms^11^. The L33P substitution located in the *PSI* domain is responsible for the HPA-1 system, clinically the most important one in Caucasian populations. A third form with a Valine in position 33 of β3 was also studied^12^. Although the 3 variants mostly shared common conformations, the P33-β3 variant presented a higher mobility and specific conformations of *IEGF*-*1*, *IEGF-2*, and *PSI* domains. The L33V substitution mainly displaced a dynamic equilibrium between common structures that could explain a variable reactivity of different anti-HPA-1a sera with the two β3 forms.

Using new strategies in MD, we studied the effect of 7 variants of the α_IIb_ *Calf-1* domain known to impair α_IIb_β_3_ integrin expression in GT. Unexpected long-range structural effects of mutations were discovered. Furthermore, our results raised the question about a possible role of these allosteric effects in the impaired α_IIb_β_3_ expression.

## Methods

### Structural data

The α_IIb_ *Calf-1* domain was extracted from a 2.55 Å resolution crystal structure of the α_IIb_β_3_ integrin (PDB code 3FCS^9^). *Calf-1* is a domain of 141 residues [positions 603–743] (see supplementary videos 1 and 2). It is a mainly-beta sandwich protein with an immunoglobulin-like topology as described in CATH database (CATH number: 2.60.40.1510, http://www.cathdb.info/version/latest/domain/3fcsA03)^13^. Some missing atoms in side chains of residues 667 and 668 were completed using Modeller software v.9.14^14, 15^. The seven GT aa substitutions were introduced in *Calf-1* structure by *in-silico* mutagenesis using PyMOL software^16^ and the SCWRL method^17^. The effects of all mutations were studied exclusively.

### Molecular Dynamics

MD simulations were done using GROMACS 4.5.7 software^18^ with the OPLS-AA force-field^19^. WT and variant forms of *Calf-1* were soaked in a rhombic dodecahedral simulation box with TIP3P water molecules and neutralized with Cl^-^ ions. The MD protocol had been used in our previous works^11, 12^. After 1 nsec of equilibration (with position restraints on the protein), each system was simulated through 11 independent dynamics for a total of 850 nanoseconds (5 × 50 nsec + 6 × 100 nsec). Molecular conformations were saved every 100 psec for downstream analysis. The first 5 nsec of each MD simulation were discarded as the residues at the extremities. Trajectory analyses were done with the GROMACS software, in-house Python and R scripts. Root mean square deviations (RMSD) and root mean square fluctuations (RMSF) were calculated on Cα atoms only. Residues interactions were analysed using the online tool PIC (Protein Interactions Calculator)^20^.

### Protein Blocks analysis

Protein Blocks (PBs) are a structural alphabet composed of 16 local prototypes^21^. Each specific PB is characterized by the φ, ψ dihedral angles of five consecutive residues with each PB assignment focused on the central residue. Obtained through an unsupervised training approach and performed on a representative non-redundant databank, PBs give a reasonable approximation of all local protein 3D structures^22^. PBs are very efficient in tasks such as protein superimpositions^23^ and MD analyses^24^. They are labelled from *a* to *p*: PBs *m* and *d* can be roughly described as prototypes for α-helix and central β-strand, respectively. PBs *a* to *c* primarily represent β-strand N-caps and PBs *e* and *f* representing β-strand C-caps; PBs *a* to *j* are specific to coils; PBs *k* and *l* to α-helix N-caps while PBs *n* to *p* to α-helix C-caps. PB^25^ assignment was carried out using our PBxplore tool at GitHub^26^.

PB assignments are done for each residue of the *Calf-1* domain and over every snapshot extracted from MD simulations. The equivalent number of PBs (*N*_*eq*_) is a statistical measurement similar to entropy that represents the average number of PBs for a residue at a given position. *N*_*eq*_ is calculated as follows^22^:

1 $$Neq=\exp (-\sum _{x=1}^{16}{f}_{x}\,\mathrm{ln}\,{f}_{x})$$

where, *f*_*x*_ is the probability of PB *x*. A *N*_*eq*_ value of 1 indicates that only one type of PB is observed, while a value of 16 is equivalent to a random distribution. To underline the main differences between the wild-type (WT) and a variant for each position, Δ*N*_*eq*_ value is computed. Δ*N*_*eq*_ is the absolute difference between corresponding *N*_*eqs*_.

However, a same Δ*N*_*eq*_ value can be obtained with different types of blocks in similar proportions. To detect a change in PBs profile, a Δ*PB* value was calculated. It corresponds to the absolute sum of the differences for each PB between the probabilities of a PB *x* to be present in the WT and the variant forms (*x* goes from PB *a* to PB *p*). Δ*PB* is calculated as follows:

2 $${\rm{\Delta }}PB=\sum _{x=1}^{16}|({f}_{x}^{WT}-{f}_{x}^{\mathrm{var}})|$$

where, *f* ^*WT*^_*x*_ and *f* ^*var*^_*x*_ are the percentages of occurrence of a PB *x* in respectively the WT and the variant forms of *Calf-1* structures. A value of 0 indicates perfect PBs identity between WT and variant, while a score of 2 indicates a total difference.

### Calf-1 variants

The seven *Calf-1* domain variants studied herein were involved in GT and are reported in the GT database (https://sinaicentral.mssm.edu/intranet/research/glanzmann). Variants L653R^27^, L721R (only reported in the GT database), L721V^28^, R724P^28, 29^, R724Q^30^, and P741R^29^ severely impaired α_IIb_β_3_ expression (less than 5%) while variant C674R^28, 30–32^ allowed a 10% residual expression (type I and II GT^33^).

## Results

### Structural analysis of the Calf-1 domain

**Figure 2:**
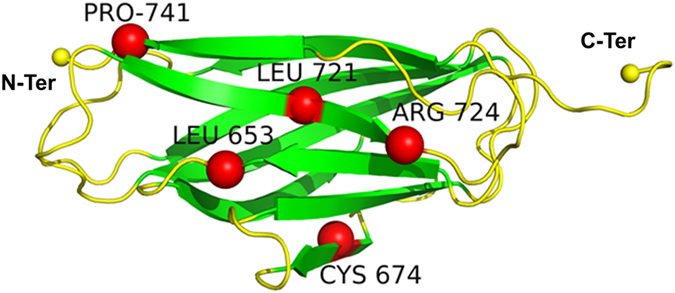
Ribbon model of the *Calf-1* domain showing the location of the studied variant residues. β-strands are coloured in green and loops in yellow. Variant residues are identified as red balls. N-ter and C-ter ends are shown as yellow balls.

**Figure 3:**
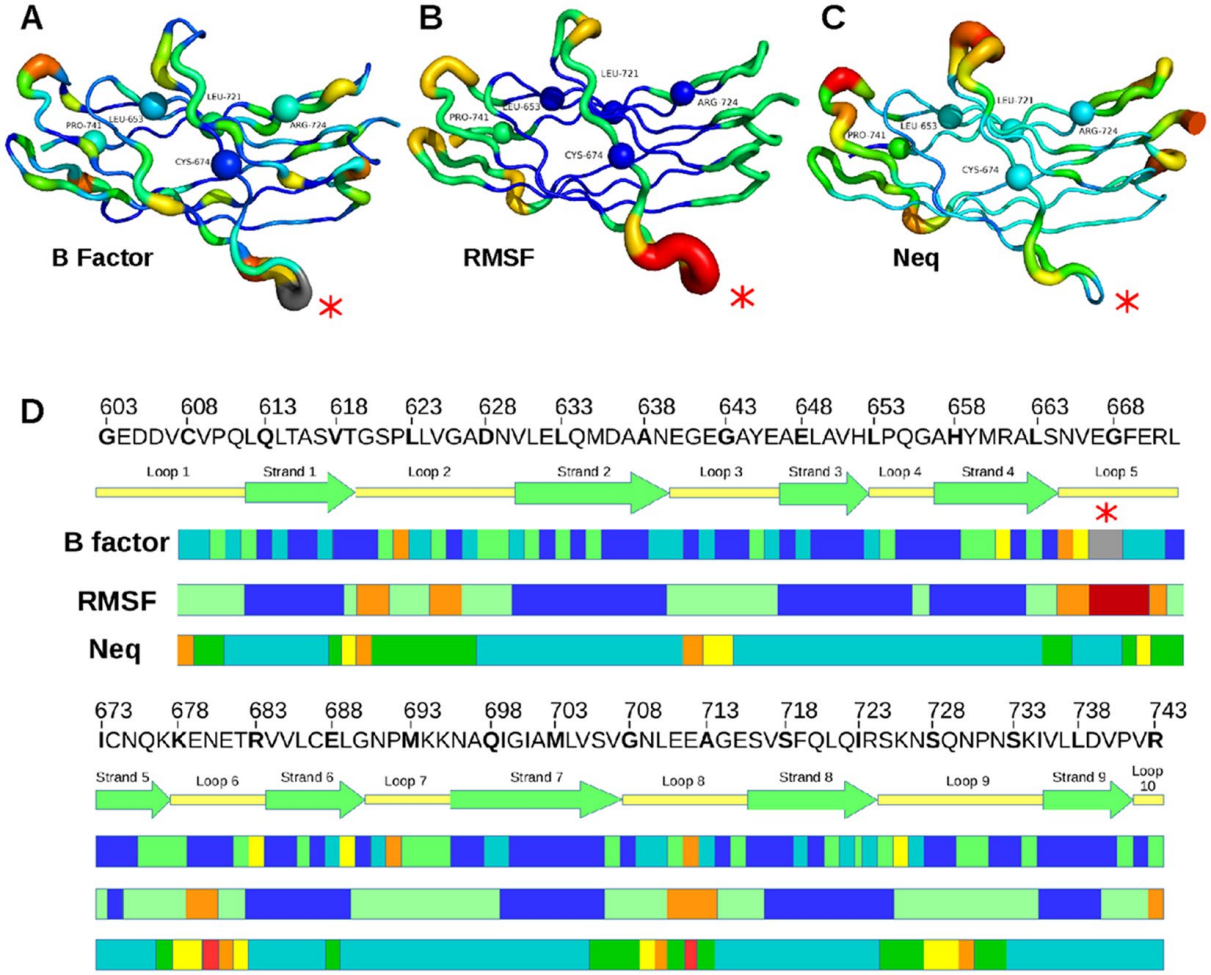
Comparison of the protein flexibility of *Calf-1* through different metrics. 3D structures of *Calf-1* domain represented through (**A**) B-factor values, (**B**) RMSF values, and (**C**) *N*_*eq*_ values. Local structure is ranked from rigid (thin blue line, a value of 0.0) to flexible (thick red line, a value of 4.0). Residues with completed missing atoms are in grey in the B-factor cartoon (**A**). (**D**) The *Calf-1* amino acid sequence is placed in regard to its secondary structures assignment and to protein flexibility according to the B-factor, the RMSF or the *N*_*eq*_ values. Blue, green, yellow, orange and red colours scale the structure from rigid to flexible. The loops are: loop 1 (size: 9, positions 603–611), loop 2 (size: 10, positions 620–629), loop 3 (size: 7, positions 640–646), loop 4 (size: 4, positions 653–656), loop 5 (size: 8, positions 665–672), loop 6 (size: 6, positions 678–683), loop 7 (size: 6, positions 690–695), loop 8 (size: 8, positions 708–715), loop 9 (size: 11, positions 725–735), and loop 10 that begins at position 742.

*Calf-1* domain extends from residues 603 to 743 of the α_IIb_ integrin subunit. This domain is an all beta structure adapting an Immunoglobulin-like Beta-sandwich fold^13^ with 9 consecutive β-strands connected by 8 loops (Fig. 2, the loops position is provided in Fig. 3D). Loops 1 and 10, located at the N- and C-terminals of *Calf-1* connects it with N-ter *Thigh* and C-ter *Calf-2* domains, respectively. RMSD from all MD simulations reach a steady state at 50 nsec (Figure S1) that is maintained in longer runs of 100 nsec indicating stable and reproducible independent dynamics.

According to the high B-factor values obtained from crystallographic data, loops 2, 3, 4 and 5 are the most flexible regions of *Calf-1* (Fig. 3A). Residues 622, 643, 710 and residues 667/668 (of loop 5 that contains missing atoms) presented the highest B-factor values in their respective loops. On average β-strands are more rigid than loops^34, 35^, although some of their residues represent relatively high B-factor values. In our study, the B-factors seemingly escape the influence of crystal packing contacts. However, it is known that B-factors are strongly influenced by the crystal packing of the structure^36^. Some protein moieties very flexible in solution might seem to be rigid only because they are involved in the solid-state packing.

RMSF values computed from MD simulations measure the mobility of each residue around its median position in the structure and allow assessing protein flexibility (Fig. 3B). High RMSF values are often associated with loops and sometimes with C-ter of β-strands. As defined by high RMSF values, loop 2 (residues 619–620 and 625–626), loop 5 (residues 665–671) and loop 8 (residues 711–713) are flexible regions, with loop 5 being the most flexible. The rest of the structure is more rigid. RMSF and B-factors values are correlated for loops 2, 5 and 8 (Fig. 3D). Some points are noteworthy: (a) the limits of flexible positions can show some little differences between RMSF and B-factor and (b) loop 3 is associated to high B-factor but low RMSF values although it binds a Ca^2+^ (not included in MD simulations) in the crystal structure. Similar correlation between B-factor and RMSF values had been previously reported^37^.

**Figure 4:**
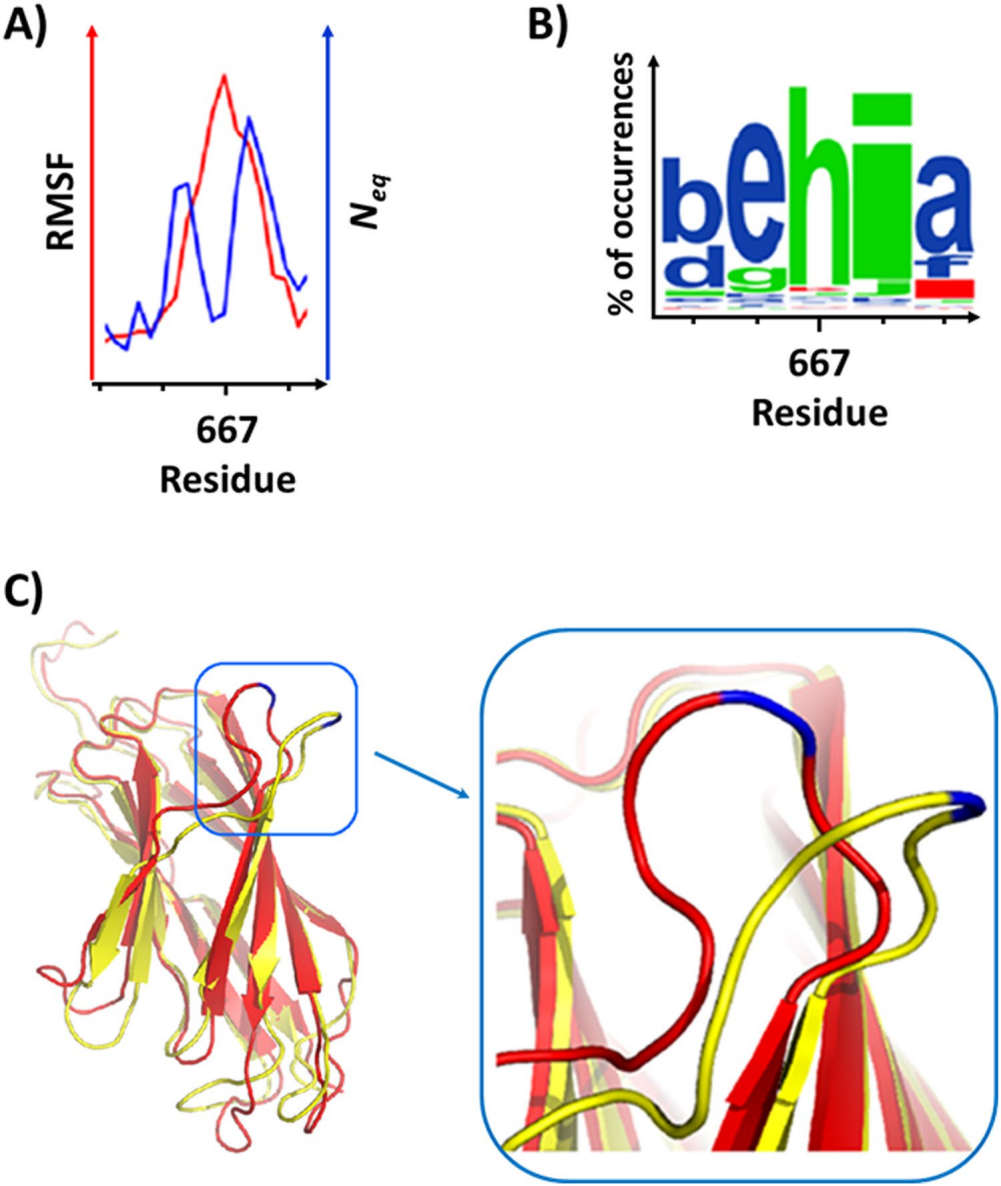
Local rigid conformation in a deformable loop, low *N*_*eq*_ versus high RMSF. (**A**) Superimposed RMSF and *N*_*eq*_ values (red and blue curves respectively) from residues N665 to G668, (**B**) The WebLogo^49^ indicates the frequency of occurrences with respect to the PBs adopted (size of the letter) by a residue in MD simulations. Here, residues V666 to F669 mainly adopted the PBs profile “e*hia”* corresponding to low *N*_*eq*_ for them. (**C**) 3D model of the *Calf-1* domain and the frame magnified of two adopted by the loop conformations (red and yellow worm-lines) carrying the residue E667 (in blue) that keep a rigid structure relative to the mobile loop.

Figure 3B–D indicate a good correlation between RMSF and *N*_*eq*_ values. Indeed, highest *N*_*eq*_ values are associated to flexible regions (as defined by B-factor and RMSF) with residues K678-T682 (loop 6) and N709-E712 (loop 8), but also with T619 (loop 2). Expectedly, some regions can show higher *N*_*eq*_ for some residues; G641-G643 (loop 3) and S728-N730 (loop 9). On the other hand, highly flexible region can also represent high local rigidity in terms of PBs, for instance, residues V666-F669 and E670 in loop 5 (Figs 3C and 4). Direct comparison of RMSF and *N*_*eq*_ values (Fig. 4A) clearly shows that E667 represents a high RMSF but a low *N*_*eq*_. This can be explained by its PB distribution (Fig. 4B): E667, G668 and F669 representing the highest RMSF values (and also B-factors), mainly adopted the PB sequence “*hia”* with respective occurrences of 86.2, 82.9 and 61.6%. A series of PB “*hia”* is a classical loop conformation but this region (in blue rectangle on Fig. 3C) maintains a single conformation and is not really flexible. This apparent discrepancy can be explained by the insertion of the rigid stretch E667-F669 in a larger flexible (or more precisely deformable) loop N665-L672. Interestingly our results revealed that a locally rigid aa stretch (few possible conformations/low *N*_*eq*_) can be a part of a large mobile loop involved in the global structural motions of the protein (high RMSF).

Overall, our results showed a good correlation between experimental data (B-factor), RMSF and *N*_*eq*_ obtained from MD simulations. Although some discrepancies did exist, they are explained by local structure singularities. As expected in an all-Beta domain, rigid β-strands are linked by flexible loops.

The selection of OPLS-AA force field was made in regards to our previous works on integrins^11, 12^. To assess the effect of the force field, simulations were also performed on WT with Gromos 46a7 and Gromos 54a7. For the latter, 11 independent simulations were performed with the protocol used with OPLS-AA. No significant global or local differences were observed.

### Structure comparisons between GT variants and WT Calf-1

The α_IIb_β_3_ integrin was cut into compact structural domains through Protein Peeling^38^ that correlate the delineations found in literature^9^. As shown in Fig. 2, the variant residues studied are mostly located at β-strands presenting low flexibility with the exception of residue 653 localized near the β-strand 3 C-ter.

As for the WT system, the 7 variants were studied with 11 independent MD simulations performed to a complete timing of 850 nsec and with parameters similar to Jallu *et al*.^12^. Each system reached a plateau after 5 nsec with an average RMSD of 2 Å (beginning of loop 1 and end of loop 10 excluded). All energetic and geometric parameters showed a good evaluation for the 77 different simulations used in this study, e.g. no clashes found. The *Calf-1* domain stayed consistent during the whole dynamics.

**Figure 5:**
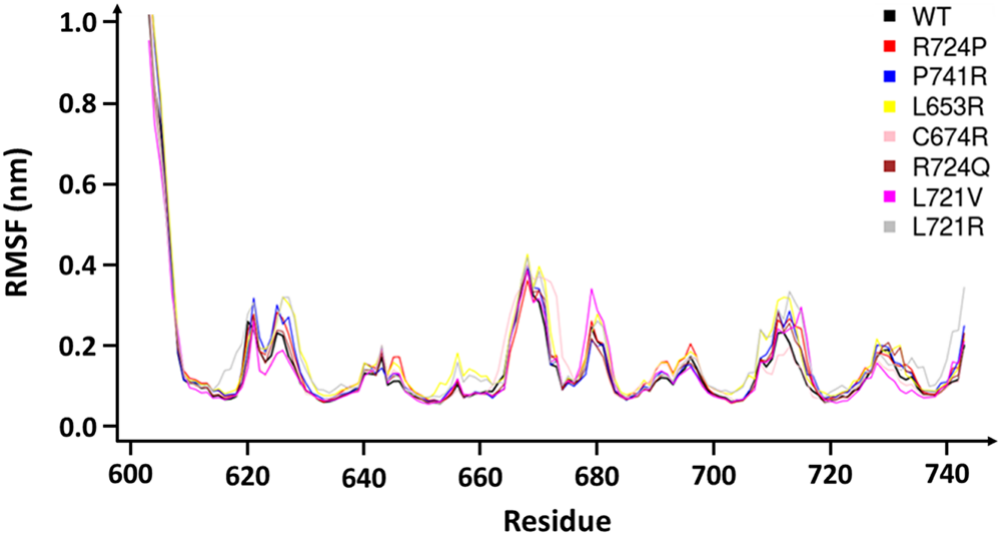
*Calf-1* RMSF of the different systems. By comparison, *Calf-1* variant structures mainly behaved like the WT form (black curve). The noisy peaks for the N-ter first residues were discarded in the majority of the analyses since, in nature they lay at conjunction to the neighbouring domain.

Average RMSF from each variant and the WT were comparable (Fig. 5). The most important variations observed in loop 2 (V625), loop 5 (E670), loop 8 (A713) and loop 9 (N732) did not lead to disordered patterns. Some variants showed specific higher or lower RMSF for some restricted positions like for C674R and L721V variants (Fig. 5). As seen with the WT system, RMSF can be confused with deformability. To resolve this aspect, we have used PBs analyses of the MD trajectories.

### PBs analyses revealed striking local structure alterations, but distant from the variant sites

For clarity, results are detailed for only 3 variants R724Q, L653R and C674R that are representative of all behaviours observed for the 7 variants.

### R724Q

**Figure 6:**
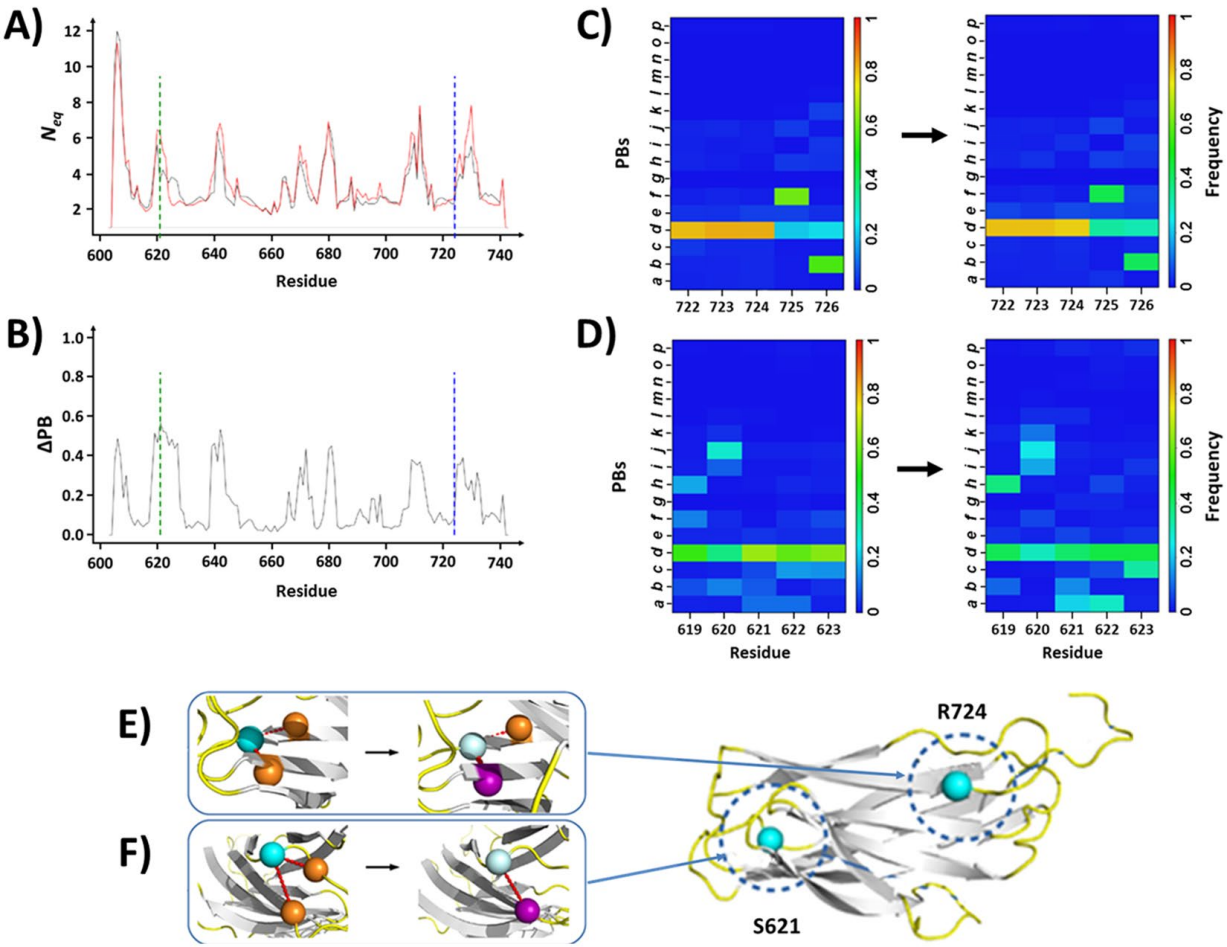
Variant R724Q. (**A**) *N*_*eq*_ values of residues from the WT system (black curve) and of the R724Q variant (red curve). Positions of the residues 724 and S621 that presented the highest RMSF alteration are respectively indicated by blue and green dots lines. (**B**) Curve of the ΔPB values computed from the difference between the two systems. (**C**) PB maps from residues 722 to 726 for the WT (left) and the variant (right) systems. (**D**) PB maps for residues 619 to 623. Color scales indicate the frequency of occurrences of the PBs in the map. (**E**) Molecular interactions made by the residues 724 in the WT and the variant systems (left and right cartoons respectively). (**F**) Molecular interactions made by the residues 621 in the WT and the variant systems (left and right cartoons respectively). Residues 724 and S621 are shown as cyan balls in the WT form, and as light cyan balls the variant. Orange balls indicate residues that conserved their interactions with the residues 724 or S621 while magenta balls correspond to residues with modified interactions. A cartoon of the *Calf-1* domain shows the respective locations of the residues 724 and 621.

This aa variation is located at β-strand number 8. In regards to the WT structure (Figs 6A and S2A), the highest *N*_*eq*_ differences are at S621 (beginning of loop 2), A644 (loop 3) and L710 (loop 8). These loops that are naturally flexible are even more so in the variant. Conversely, residues L624 to D628 have a lower *N*_*eq*_ value thus indicating that loop 2 represents a dual behaviour, with increased deformability at its beginning and enhanced stability in its C-ter part. Surprisingly, the mutant residue Q724 (β-strand 8) conserved the same *N*_*eq*_ (Fig. 6A) with a low ΔPB of 0.09 (Fig. 6B) indicating that local β-strand conformation is conserved, *i*.*e*. PB *d*.

Regarding the structure, the polar amino acid arginine contains a longer aliphatic side-chain than glutamine, an uncharged hydrophilic polar amino acid. Q724 conserves the backbone - backbone interaction with E648 as observed with R724 (β-strand 3, see Fig. 6E). Beside, Q724 lost the ionic bond and the side chain - side chain interactions with E648 but made new hydrogen bonds through side chains interactions with E722. This showcases a classic example of structural compensation that maintained the local conformation of the residue through different interactions.

The highest Δ*N*_*eq*_ (2.71) that is also associated with the highest ΔPB (0.57), is observed for S621 (Fig. 6A,B). S621 is located at the opposite side of the domain in reference to residue 724 (Fig. 6E). In the variant structure, S621 mostly remained in a PB *d* (*i*.*e*., β-strand) conformation with however, a decreased frequency of occurrences. Besides, downstream P622 and L623 presented some lost conformers with increased frequencies of PBs e and h respectively. Very few typical backbone - backbone interactions of S621 with L623 and backbone - side chain interactions with N629 are replaced by a single bond between side chains with N629. Adding to this high mobility, S621 did not do consistent and sustainable interactions. This behaviour is amplified in the Q724 variant and the most stable residue S621 in a naturally flexible region (loop 2), became one of the most deformable position.

### L653R

**Figure 7:**
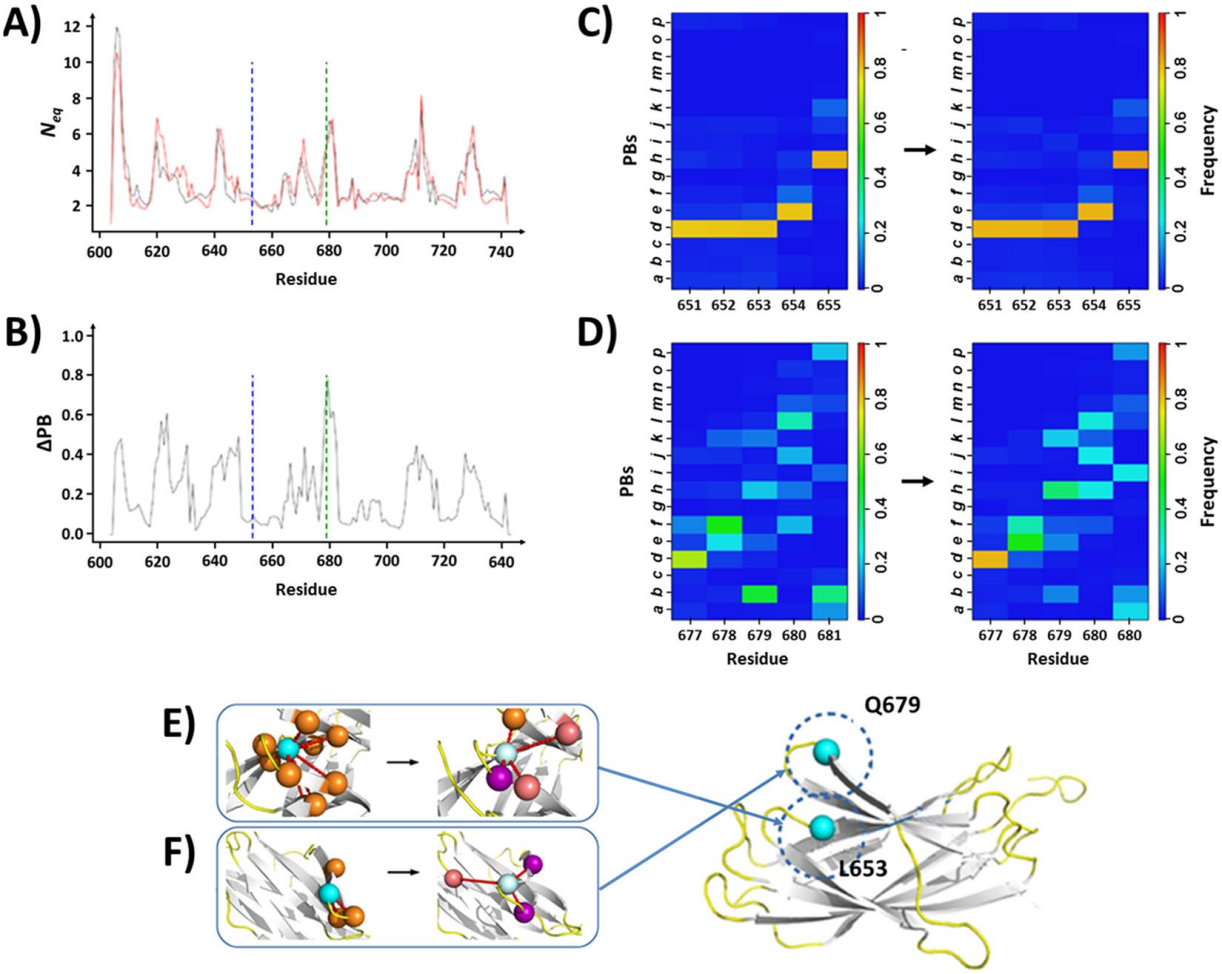
The variant L653R. Panels (A and B) respectively show the *N*_*eq*_ and the ΔPB curves of the L653 (WT) and R653 (variant) systems. Panels (C,D) respectively show the PB maps of residues 651 to 655 and 677 to 681 with the WT at the left and the variant form at the right. (**E**) Molecular interactions made by the residues L653 or R653 and (**F**) by E679. For colour scales and residue presentation, see the legend of Fig. 6.

This GT variant results from a L653R substitution in loop 4. The highest *N*_*eq*_ variations (Figs 7A and S2B) affected residues G620-P622 (loop 2), V630-L631 (β-strand 2), E646 (loop 3), R671 (loop 5) and L710 (loop 8). As observed with the R724Q substitution, residues G620-L623 gained slightly more flexibility. Conversely, residues L624-D628 showed increased flexibility but with a limited impact (ΔPB 0.23 on average) on the most frequent PBs (PB *e* for L624, *h* for V625 and *i* for G626 in Fig. 7B).

The mutated residue in position 653 (loop 4) was not subjected to any *N*_*eq*_ modification. It conserved a strong local structural stability (Fig. 7C) similar to its direct environment. The PB series at this position “*dddeh”* is even slightly more common in the variant than in the WT (64% and 59%, respectively). In the R653 variant, the 8 hydrophobic bonds of L653 disappeared in favour of new interactions between the R653 backbone and A657 and E676 side-chains (Fig. 7E). The backbone – backbone interaction with R683 was conserved. The mutation zone showed no conformational change as the loss of important specific interactions were partly compensated by new ones. Over 9 original interactions only 1 is conserved while 3 are created.

Q679 (loop 6) is a very interesting case where Δ*N*_*eq*_ was negligible while the ΔPB was the highest (0.78). The most frequent PB *b* (N-cap of β-strand) was replaced by a PB *h* (loop structure) in regards to their frequency of occurrences (Fig. 7D). Hydrogen interactions with T682 and K677 are retained but the backbone - backbone interaction with E681 was lost and replaced by side chain and ionic side chain interactions with R724 in loop 9. In the variant structure, this region has high fluctuations in PBs, mainly associated to loops that even affected the C-ter of the β-strand 5 located the above loop 9.

### C674R

**Figure 8:**
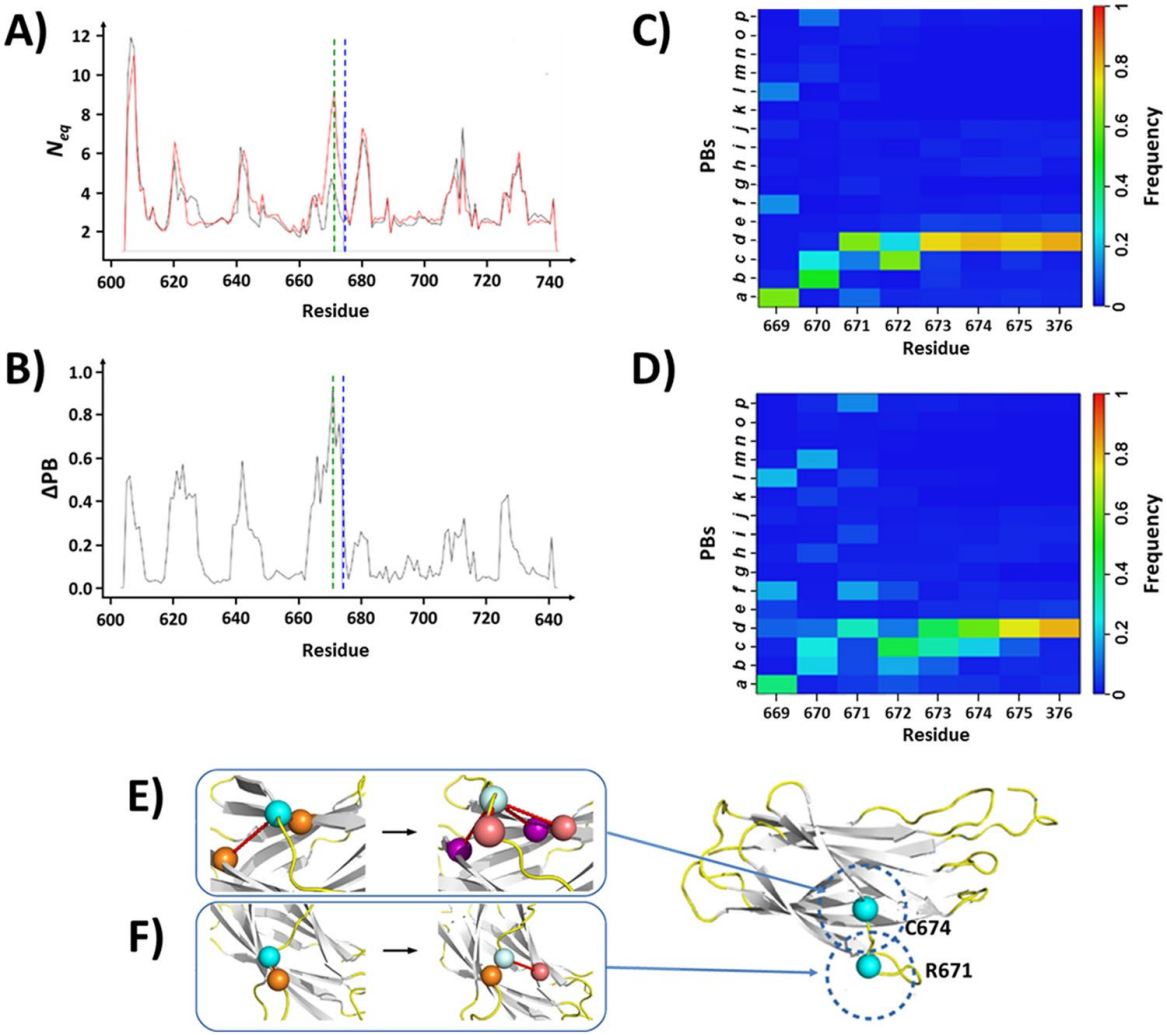
The variant C674R. Panels (A and B) respectively show the *N*_*eq*_ and the ΔPB curves of the C674 (WT) and R674 (variant) systems. Panel **C** show the PB maps of residues 669 to 676 for the WT (above) and the variant forms (below). (**D**) Molecular interactions made by the residues C674 or R674 and (**E**) R671. For colour scales and residue presentation, see the legend of Fig. 6.

This variant is associated with a C674R substitution in β-strand 5. We observed a variation of *N*_*eq*_ profile (Figs 8A and S2C) similar to that occurring in the R724Q substitution (see previous section). Loop 2 presented the same increased deformation at its beginning (S621), followed by a stiffening in its centre (residues L624-D628). The same PB series “*ehiac”* (L624-D628) is found in greater proportion than in the Q724 and R674 variants, reinforcing the local stiffening of the loop in this region.

The main destabilization was far upstream of residue 674 (Fig. 8C). With the C674R substitution, the residue 674 not just lost its covalent disulphide bond with C687 located at the end of β-strand 6, but also its aromatic interaction with Y659 in β-strand 4 (Fig. 8D). However, the mutated R674 made an ionic bond with E688 located at end of β-strand 8 that strengthened a backbone – backbone interaction. The 80% frequency of PB *d* (the highest) in WT decreased to 49% in the variant. Surprisingly, N675 and Q676 located downstream the substitution remained structurally stable with similar PB occurrences.

The highest *N*_*eq*_ variation affected R671 as shown by the strongest Δ*N*_*eq*_ (5.02) and ΔPB (0.91). The side chain of R671 is mainly exposed at the domain surface and forms a single ionic interaction with the neighbouring E670, like in WT. But in the variant conformation, the R671 side-chain can occasionally turned toward loop 8 to make ionic side chain interactions with E688 (Fig. 8E). The frequency of PB *d* (the highest) drastically decreased in the variant leading to an increased disorganization of the neighbourhood.

Experimentally, the C674R mutation severely impaired the α_IIb_β_3_ complex expression with only 10% expressed at the surface of the patient’s platelets and transiently transfected CHO cells^31^. The C674R mutation did not impair pro-α_IIb_ synthesis but affect the stability of the complex that is not correctly matured and/or expressed at the cell membrane.

### Other variants

**Figure 9:**
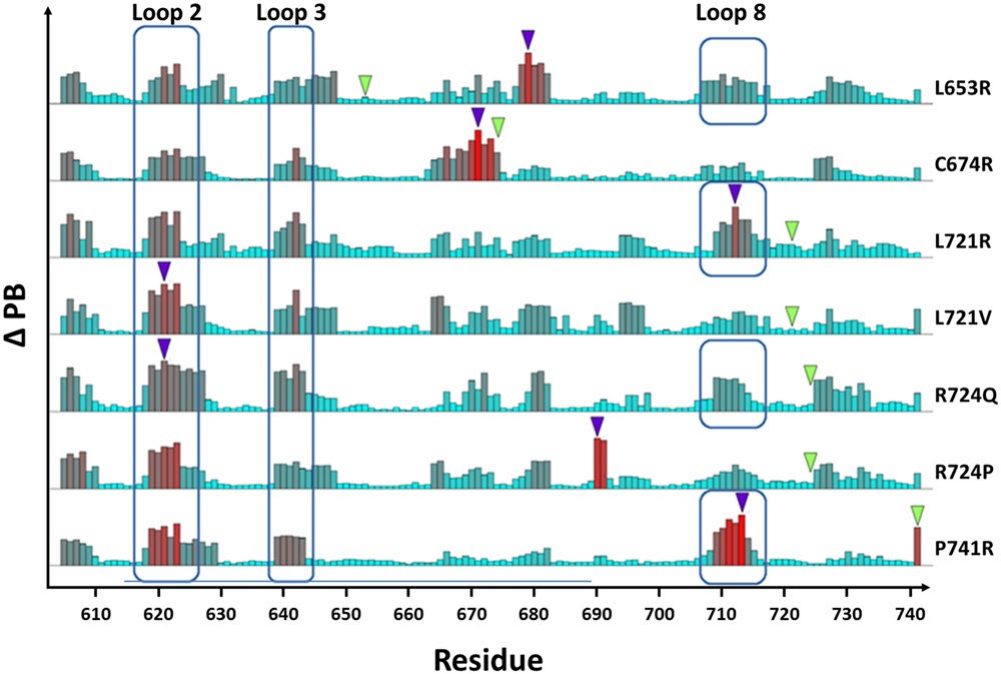
Comparative study of the ΔPB values for all WT-variant pairs. Histogram schema presenting the ΔPB value computed for each residue position (abscissa) from each variant and the WT systems. Green triangle indicates the aa variation position while the purple one position shows the position of maximal ΔPB. Residues from loops 2, 3 and 8 presenting common high ΔPB for all variants are boxed.

These MD simulations on *Calf-1* domain allowed demonstrating more or less pronounced structural changes depending on the variants under study. The C674R and P741R variants presented conformational changes at the mutated site. In the case of the C674R substitution, the resulting loss of the disulphide linkage relaxes the structure and introduces significant structural alterations (Figs 7 and 8). The proline is the aa known to cause the most drastic change in conformations^39^. Indeed, the P741R substitution (Fig. 9) inverse the PB profile going from 55% of PB *d* (β-strand) and 29% of PB *f* (C-cap of β-strand) to 24% of PB *d* and 59% of PB *f* (Figure S3). This case was associated with a low Δ*N*_*eq*_ (0.15, Figure S4A) while the ΔPB was high (0.70). In P741R substitution two hydrophobic interactions were lost and R741 formed ionic and side chain - side chain interactions shortening the β-strand. Note that residue 741 is located at two residues from the C-term of *Calf-1* and is normally in contact with the *Calf-2* domain. Thus, the absence of the neighbouring domain in our MD simulations can impact our observations. To solve this problem, MD simulations of combined domains are currently under study.

In the remaining 5 variants studied, compensation mechanisms were also observed. Most interactions formed by WT residues are replaced by new ones, allowing conservation of the local structure. Surprisingly, regions displaying significant changes (high ΔPB) are distant from substitution sites without any contact/interaction with the substituted aa. These regions contribute towards increasing the deformability and are usually located at interfaces adjacent to neighbourhood *β-propeller, Calf-2* or *Thigh* domains. These results depict changes resulting from substitutions in distant regions suggesting long-range mechanism to be at play.

### Different variants with common mutation sites

*L721R and L721V* showed quite different results (Fig. 9). Compared to L721R, the L721V substitution had very little impact on RMSF, apart for the end of loop 8, a highly flexible region. This is particularly true for E712 (loop 8), whose Δ*N*_*eq*_ were respectively of 3.33 and 0.39 in R721 and V721 variant forms (Figures S4B and S5). Residue 721 and E712 are remote (separated by β-strand 8 and a part of loop 9), and E712 can interact with the β-propeller domain. Loop 8 and especially E712 are already naturally mobile. Locally, V721 also presented a lower impact than R721 that has a higher overall Δ*N*_*eq*_. These observations sound logical as Leucine and Valine share similar hydrophobic aliphatic side-chains (a single methyl group of difference), mitigating the impact of the L721V substitution with respect to arginine, which is positively charged.

### R724P and R724Q

The previously described R724Q substitution (see above) presented a very pronounced impact compared to others and induced the dual deformability/rigidity alterations of loop 2. This particular phenomenon is also observed in the variant R724P although contributing more stiffness to the site, owing to the backbone structure of proline (Fig. 9). In both cases, the mutated residue did not affect the local conformation (Δ*N*_*eq*_ and ΔPB are identical). The P724 variant mainly differed from the R724 WT in residues G690 (C-cap of β-strand 6) and N691 (start of loop 7, in contact with the *Thigh* domain). G690 and N691 have maximal ΔPBs of 0.79 and 0.75, and Δ*N*_*eq*_ of 2.17 and 0.85 respectively. For G690, these values are consistent with a drastic PB *j* (loop) decreased from 70% in the WT to 23% in the variant, while the occurrences increased for very rare PBs *i* and *p* (loop and N-cap of α-helix) (Figure S6). For N691, PB *c* occurrences decreased from 80% to 45%, while PB *a* reached 44%; both PBs types being associated with N-cap β-strand.

Considering the 7 variants, some *Calf-1* regions are more affected than others (see Figure S6). For instance, loop 5 had certain rigidity in terms of PBs but this area is subject to strong *N*_*eq*_ increment under influence of substitutions involved in Glanzmann thrombasthenia (e.g., case of variant C674R). Loop 8 deformability is also observed to be increased, especially in the L721V substitution. The region from the middle of loop 2 to the middle of β-strand 2 (L624-E632) is also affected, from deformability (L653R, L721R) to rigidity (C674R, R724Q).

Loop 2 (especially residues G620-P622) is particularly a remarkable region with a local variability common to all variants (Figs 8 and S6). The end of β-strand 1 has high Δ*N*_*eq*_ values ranging from 2.06 to 2.71, while ΔPB ranges from 0.54 to 0.71. A direct comparison reveals that PBs *a*, *b* and *d* frequency of occurrences in the WT decreased from 66% to 37% in the variant.

## Discussion

This study aimed at investigating the α_IIb_β_3_ integrin structure in a context of GT disorder. Previously, two MD studies of GT mutations have been performed, both with very short simulation times. The first study addresses the mutation in a calcium binding site with a single 20 ns simulation for reference and variant sequences^40^. A second study carried on the β3 S189 variant was done with a single simulation of 60 ns^41^. Hence, both these studies were very short for sampling the conformational space.

**Figure 10:**
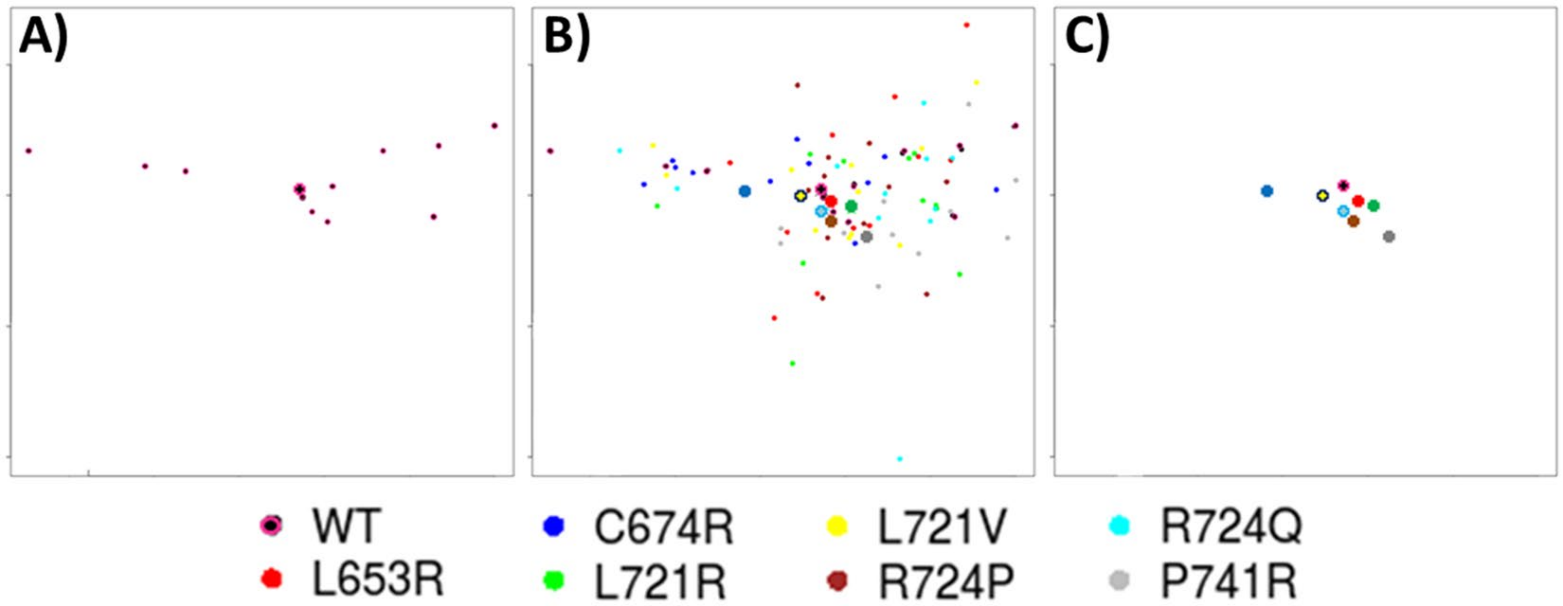
Sammon map of the MD. (**a**) The WT reference system and its barycentre, (**b**) all the variant simulations and the barycentre of each system, and (**c**) only the barycentres. The MD are shown as dots, and corresponding barycentres as plain circles.

Our team showed the impact of three distinct mutations by a static approach^10^. More recently, we have focused on the HPA-1 system characterization with MD simulations 10.8 times longer than the longest study cited herein^11, 12^. In the present work simulation times were 14.1 times longer and 11 independent simulations were done for each variant to correctly sample the conformational space. To illustrate this, Sammon maps^42^ of the 88 independent MD were done for the 8 systems (WT and 7 variants, Fig. 10). In brief, a Sammon map (SM) is a projection method, which differs from Principle Component Analysis (PCA). SM is an iterative approach that tends to conserve the distances between data within a high-dimensional space into a smaller dimensional space, often a 2D space. A major interest of SM is that it does not overweight the outliers; therefore the projection is really pertinent. SM of MD encoded in terms of PBs allowed comparing distances between MD obtained in a single or multiple systems. Considering first a single system, the limited numbers of simulations in a close vicinity to its barycentre underlined the differences between simulations as observed for the WT (Fig. 10A). Similar dispersion is obtained for each system (Fig. 10B). Two systems with variants at the same position like L721R (green dots)/L721V (yellow dots), and R724P (brown dots)/R724Q (cyan dots) can also lead to somewhat superimposed positions (Fig. 10B). Nonetheless comparing the whole set of positions (or their barycentre) clearly indicate differences in behaviour of MD of each system. It confirmed the necessity to proceed with several independent simulations otherwise the conclusions might be biased.

Sammon maps (Fig. 10C) showed that it is also possible with PB encoding to find some relationship between different systems. C674R and P741R were the most distant systems compared with the WT, while the other variants studied are closer to the reference. Interestingly the two variants of residue 721 are not close (yellow /green dots) while the variants of residue 724 (brown/blue dots) are. These examples showed that 2 variations at a same position could lead to similar or different behaviours.

Calf-1 domain is an all-β structural domain^9^. Analysis of both B-factors obtained from crystallographic data and RMSF measured from simulations showed that the more flexible regions are connector loops, as expected. The usage of three different metrics, B-factor, RMSF and PBs related values (*N*_*eq*_ and ΔPBs), provided a more complex yet comprehensive way to analyse protein flexibility locally. Comparison of these metrics revealed some interesting cases and some of these results are worth to be underlined. Loop 5 had the highest B-factor (where some atoms are missing^43^) and RMSF values. However, low *N*_*eq*_ values indicate that a highly rigid zone forms the core of the loop. This underlines interesting contexts of mobility: rigid stretch encompassed in larger deformable region leads to high mobility (high B-factor and RMSF values) while locally the loop structure did not change (low *N*_*eq*_ values). In the case of loop 2, its central residue P622 is associated to a high B-factor value, but neither high RMSF nor high *N*_*eq*_ are. It is its neighbourhood regions that would have the highest mobility. Other interesting cases are positions V625-G626 (loop 2) and E642-G643 (loop 3) that had antinomic behaviours. Both had low B-factors, even if E642 is interacting in the crystal structure with Ca^2+^ ion. While residues V625-G626 had high RMSF and low *N*_*eq*_, mimicking the loop 5 behaviour in a lesser extent, residues 642 to 643 had medium RMSF but high *N*_*eq*_. This last case is more complex: structural variations though associated to several distinct conformations (high *N*_*eq*_) were highly local, limiting the RMSF values.

These results underline the interest of *N*_*eq*_ computation in regards to classical measures and reveal the high complexity of MD thus enabling to highlight a single or only few critical residues.

The most surprising result concerns the minimal or absent structural effect observed at the aa variation site. Apart from the two very specific cases of C674R (leading to disulfide bridge disruption) or R724P (replacement by a proline) mutants, in all other studied GT variants, local conformations are maintained through new molecular interactions. Nonetheless such compensatory mechanisms cannot account for impaired αIIβ3 expression associated with the 7 GT variants tested. Another surprising result is that most important alterations are located at long distances from the mutation sites. These alterations increase mobility or deformability of the same adaptable regions (*e*.*g*. loops 2, 3 and 8) in all variants, a new concept to biomolecular structural biology as defined by R. Nussinov^44, 45^. Furthermore although RMSF profiles of the WT and the 7 variants were very similar (Fig. 5), it is particularly surprising that *N*_*eq*_ values of most loops are affected, sometimes drastically (Fig. 9). Only loops 4 and 7 presenting very low *N*_*eq*_ values in the WT form, were least affected.

The 7 variants tested under the present work severely impacted the α_IIb_β_3_ integrin expression. Using NN-Splice^46^ and GENSCAN^47^ on-line softwares, the genomic mutations involved in the aa variations studied were not predicted to induce splicing defects (Results not shown). Thence these aa variations should alter the biosynthesis of the α_IIb_β_3_ complex by structural alterations. Hence, these variant structures represent common and specific structural effects regarding the WT *Calf-1* domain (Δ*N*_*eq*_ and ΔPBs). Observed modifications rather concerns structure dynamics than large structural alterations (loss of interaction^10^) as previously seen in other studies. These results raise the possibility that current explanations of GT type I and II phenotypes regarding structure alterations might be reconsidered. In fact our results point out that structural mechanisms leading to an impaired expression of α_IIb_β_3_ integrin can be far more complex than expected. It must be noted that our study did not allow identifying which of the observed effects are responsible for the pathogenicity of the aa variations tested. Interestingly, two mutations at the same site (L721R/L721V and R724P/R724Q) can lead to similar or different effects, underlining that long-range effect depends upon the localization of the variations and also on its nature. Regardless of the effect, all variations severely impaired the expression of α_IIb_β_3_ integrin.

The expression of a functional α_IIb_β_3_ integrin at the platelet surface requires: (1) the synthesis of each subunit; (2) their association and (3) their consecutive post-translational maturation. Hence the situation can be far more complex when considering the different aspects of the biosynthesis processing of proteins. Allosteric alterations identified (or suggested) here may affect conformations of the integrin complex not described by our reference structure - the initial structure obtained from a crystallized, bent, inactivated form of α_IIb_β_3_. Interestingly, a study by Mitchell *et al*.^48^ suggested that the α_IIb_ adopts a bent form early in its synthesis process. Finally functions of other proteins like chaperones or translocon potentially involved in the synthesis process could also be affected by α_IIb_β_3_ structural alterations.

Although difficult to interpret in terms of pathogenicity our results showcased that aa substitutions identified in GT induced long-range alterations in the dynamics of the *Calf-1* domain. To explore new potential pathogenic mechanisms, we are currently addressing the role of other variants in allosteric alterations of α_IIb_β_3_ and particularly the role of neutral variants regarding the expression of the integrin α_IIb_β_3_.

## Electronic supplementary material

Supplementary information

Supplementary video 1.

Supplementary video 2.
