# Supplementary material for: In silico analysis of Glanzmann variants of Calf-1 domain of αIIbβ3 integrin revealed dynamic allosteric effect
Source: Sci Rep. 2017 Aug 14;7:8001. doi: 10.1038/s41598-017-08408-w (PMC5556033; doi:10.1038/s41598-017-08408-w)
Supplement: Supplementary file 1 — Supplementary information [file 41598_2017_8408_MOESM1_ESM.pdf]

In silico analysis of Glanzmann variants of *Calf-1* domain of $\alpha_{IIb}\beta_3$ integrin revealed dynamic allosteric effect

Matthieu Goguet[1,2,3,4,5,#], Tarun Jairaj Narwani[2,3,4,5,#], Rachel Petermann[1,5], Vincent Jallu[1,5,+] & Alexandre G. de Brevern[2,3,4,5,+,*]

[1] Platelet Department Unit, INTS, F-75739 Paris, France.
[2] INSERM, U 1134, DSIMB, F-75739 Paris, France.
[3] Univ Paris Diderot, Sorbonne Paris Cité, UMR_S 1134, F-75739 Paris, France.
[4] Institut National de la Transfusion Sanguine (INTS), F-75739 Paris, France.
[5] Laboratoire d'Excellence GR-Ex, F-75739 Paris, France.

Supplementary information

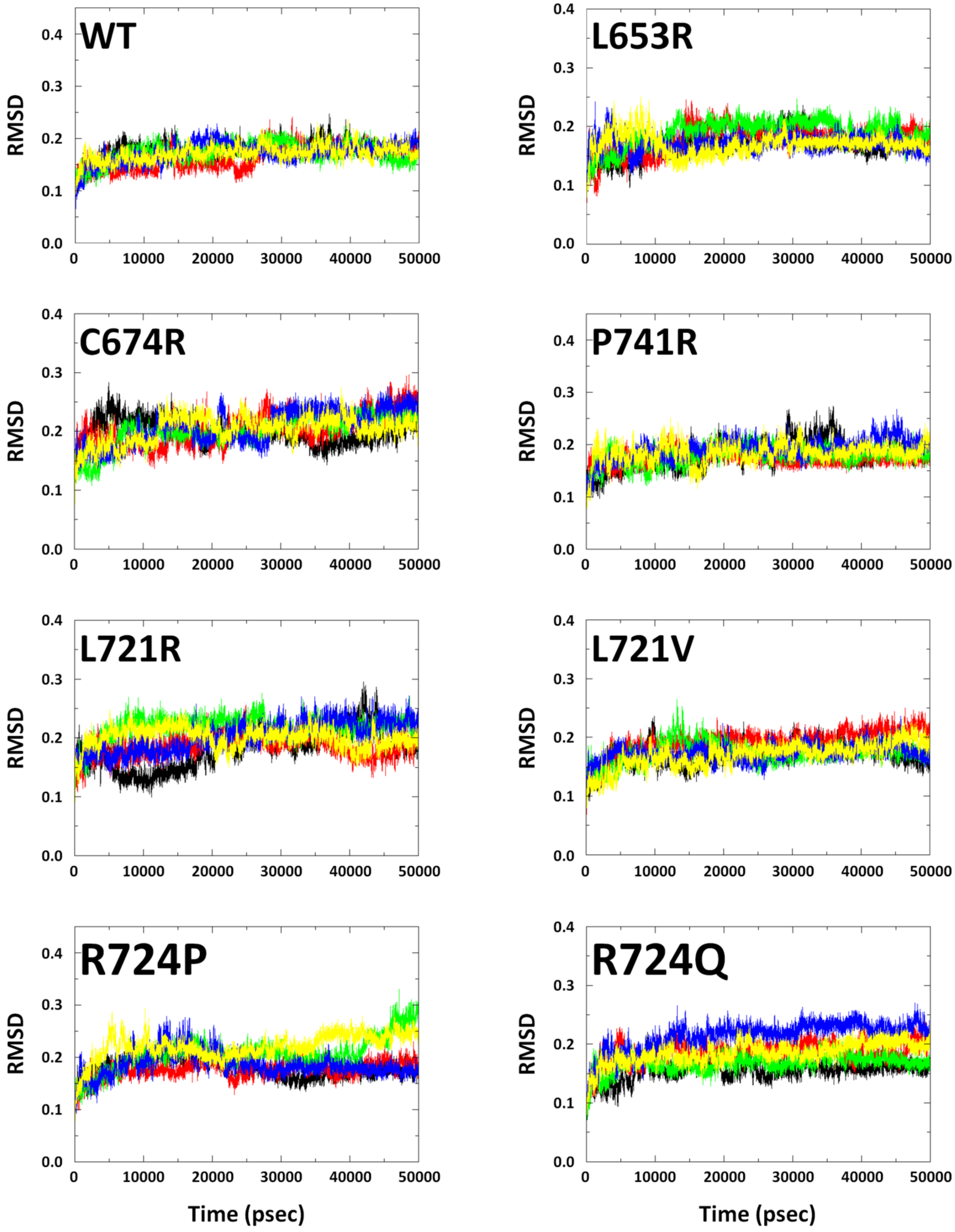

Figure S1: RMSD curves of the WT form of Calf-1 domain. Shown are curves of the 5 MD simulations performed for 50 nsec. All curves converge at 25000 picoseconds to reach a steady state.

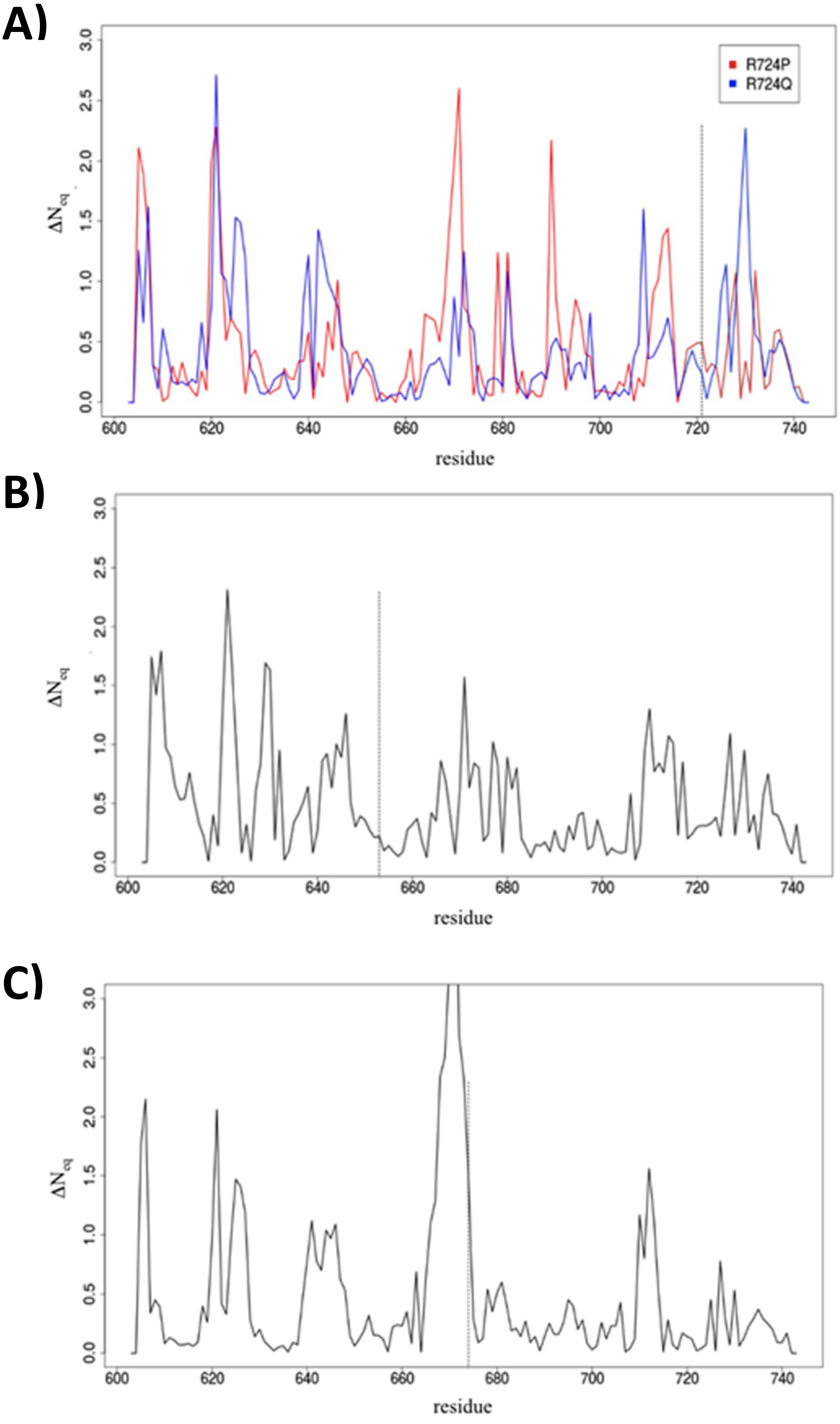

Figure S2: Difference of N_{eq}. A) For residue 724, in red R724P and in blue R724Q, B) for residue 653 with L653R and C) for residue 674 with C674R.

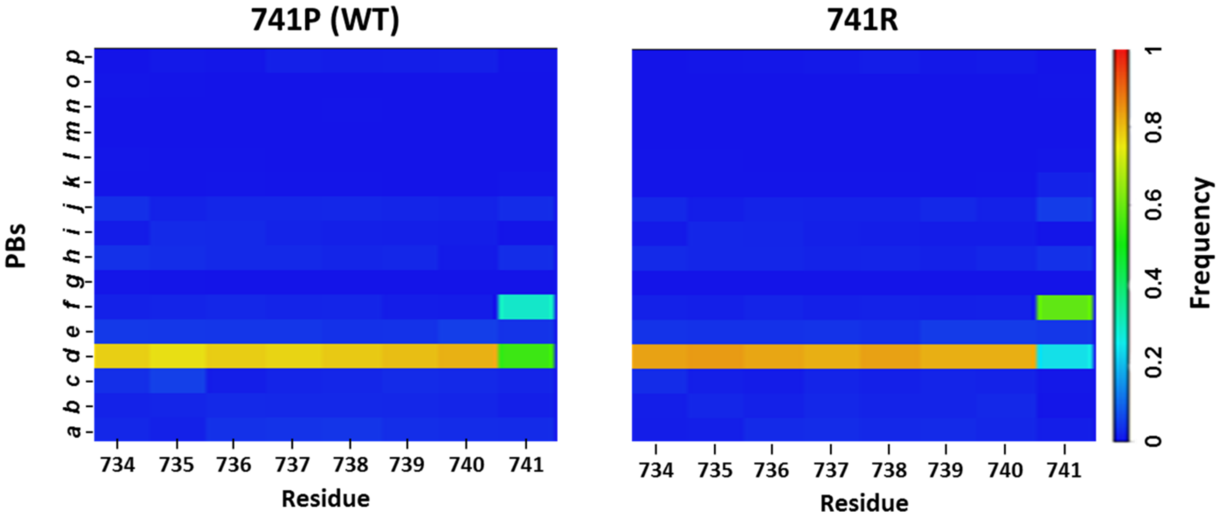

Figure S3: PB maps (residues 734-741) of 741P (WT) and 741R Calf-1 forms. Frequencies of PB *d* and *f* of residue 741 are almost inverted in WT and in variant.

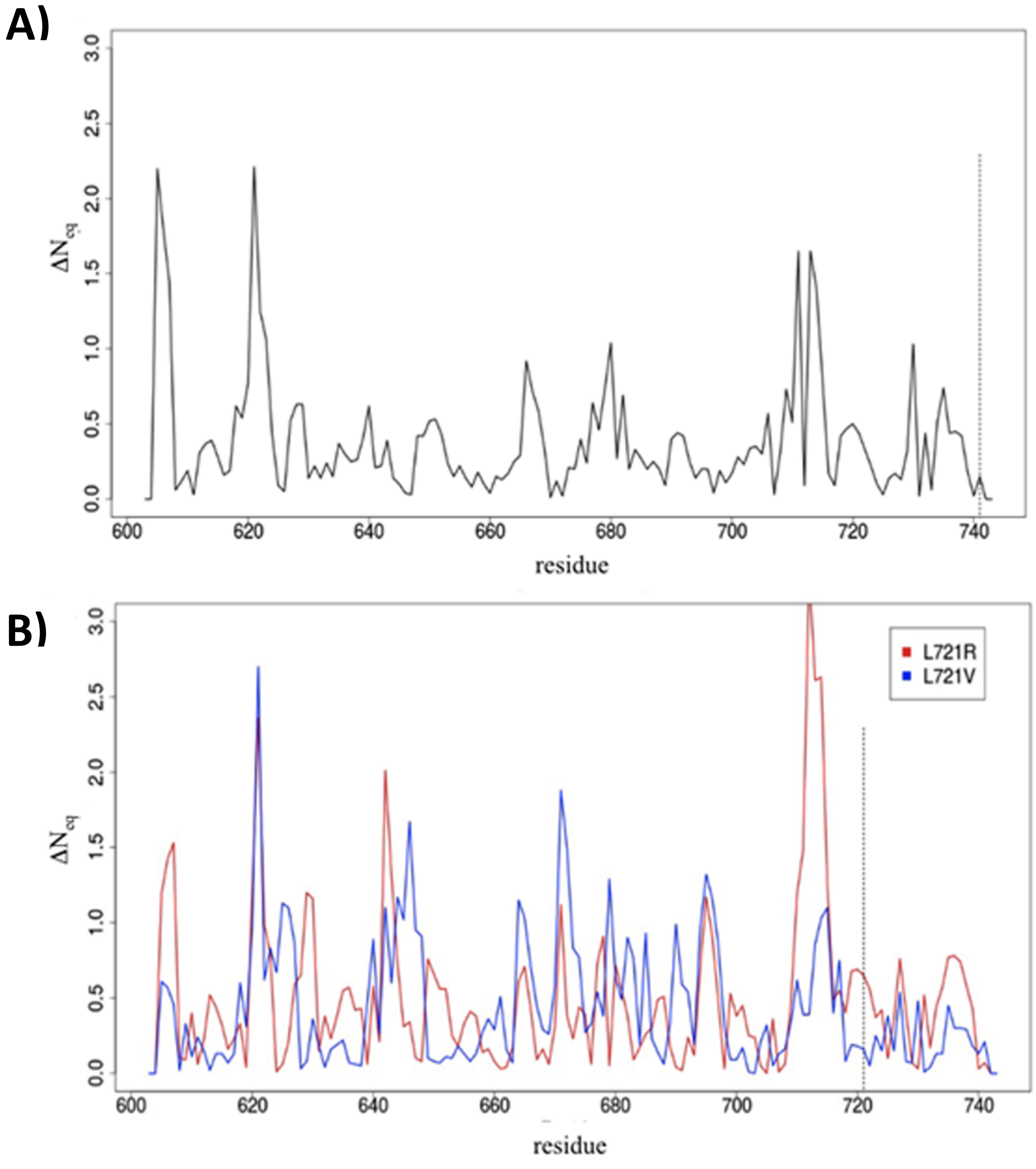

Figure S4: Difference of N_{eq}. A) For residue 741 with 741P, and B) for residue 721, in red L721R and in blue L721V.

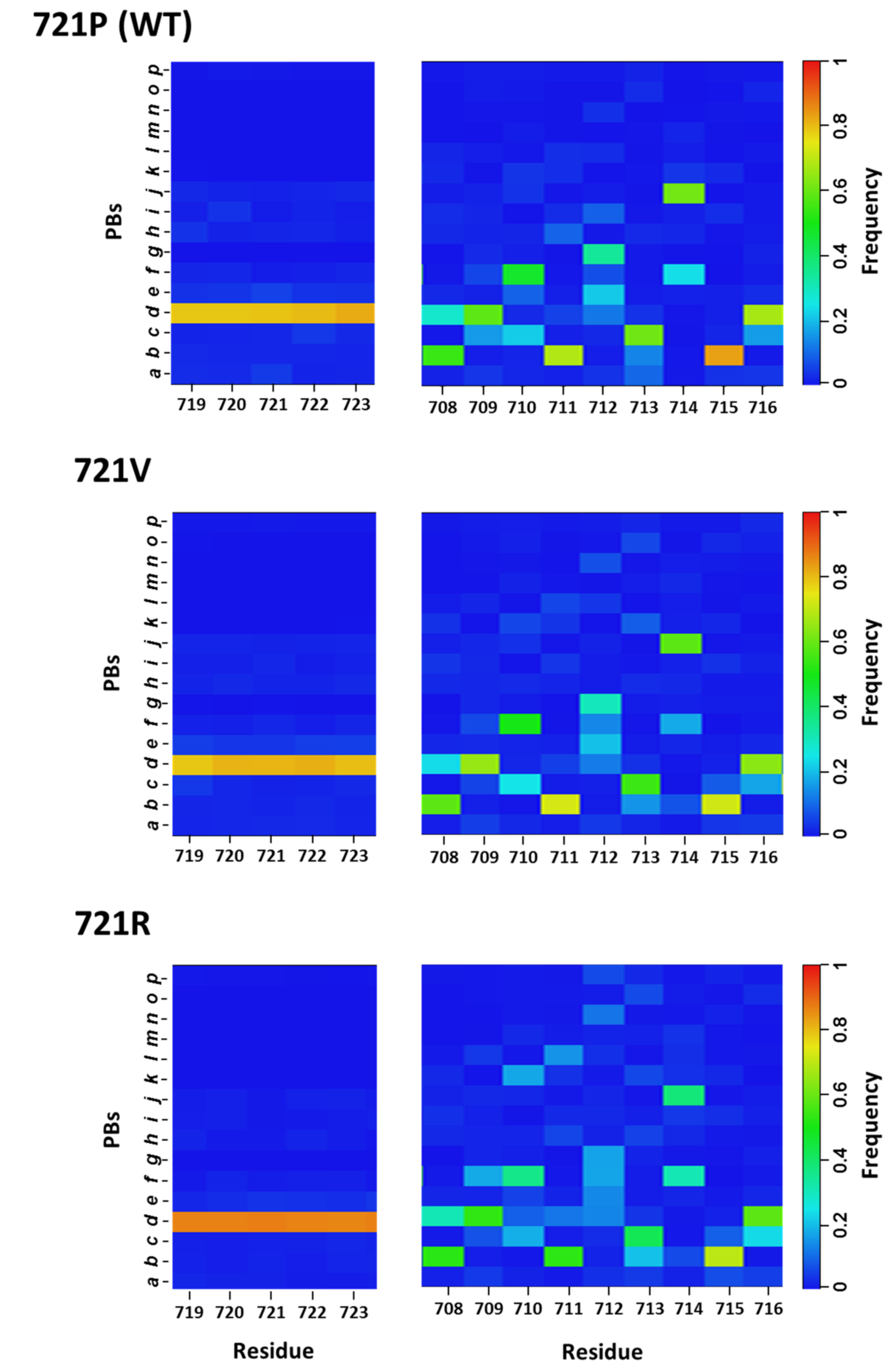

Figure S5: PB maps of 721P (WT), 711V and 721R Calf-1 forms. Shown are PB maps for residues 719 to 723 encompassing the mutation site (residue 721) (left panel) and residues 708 to 716 with residues impacted by the substitution (right panel). No significant impact is observed at mutations sites while PB profiles of residue E712 are modified in variants.

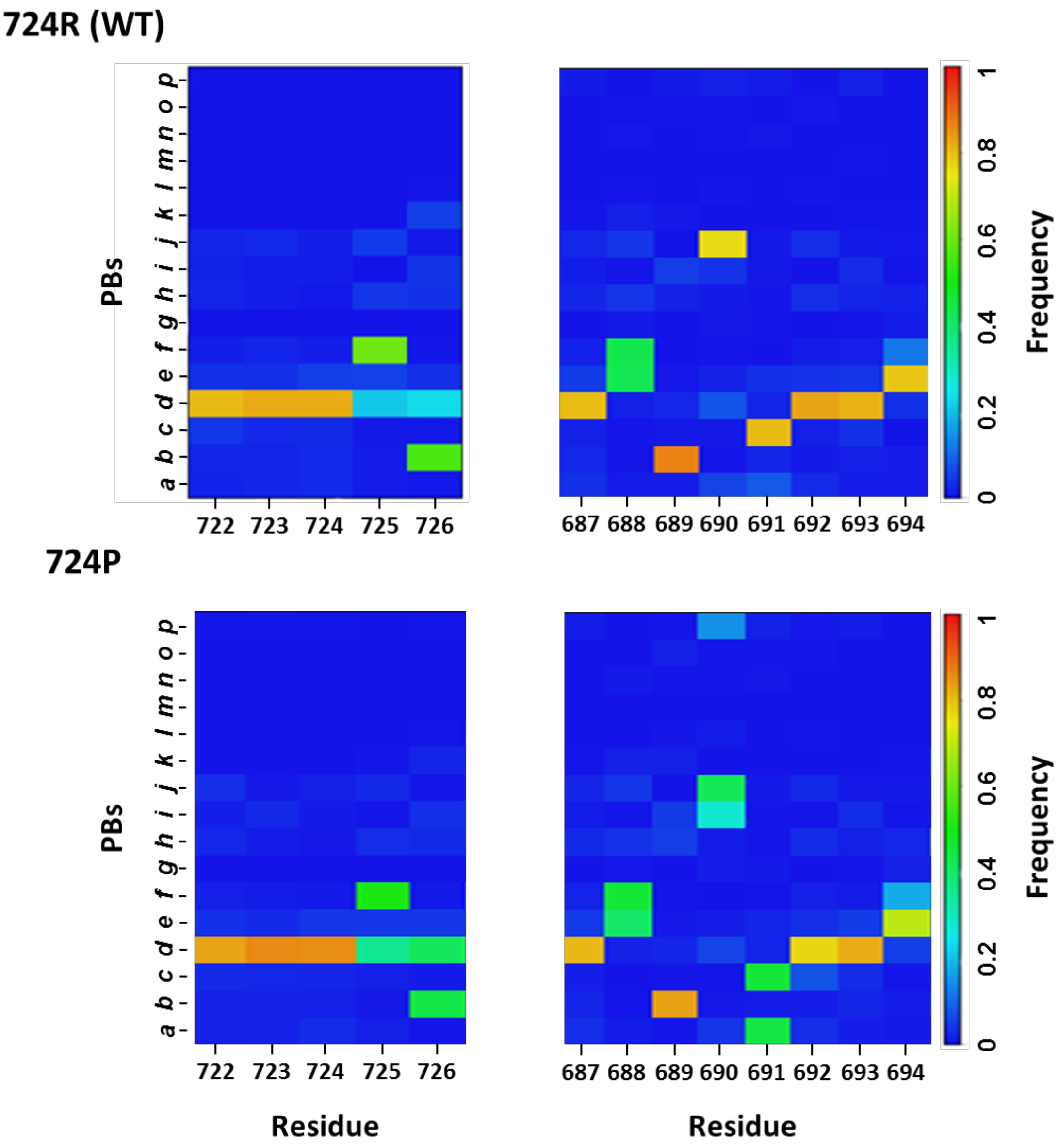

Figure S6: PB maps of 724R (WT) and 724P Calf-1 forms. Shown are PB maps for residues 722 to 726 encompassing the mutation site (residue 724) (left panel) and residues 687 to 694 with residues impacted by the substitution (right panel). PB profiles of residues 690 and 691 are impacted by the R724P substitution.

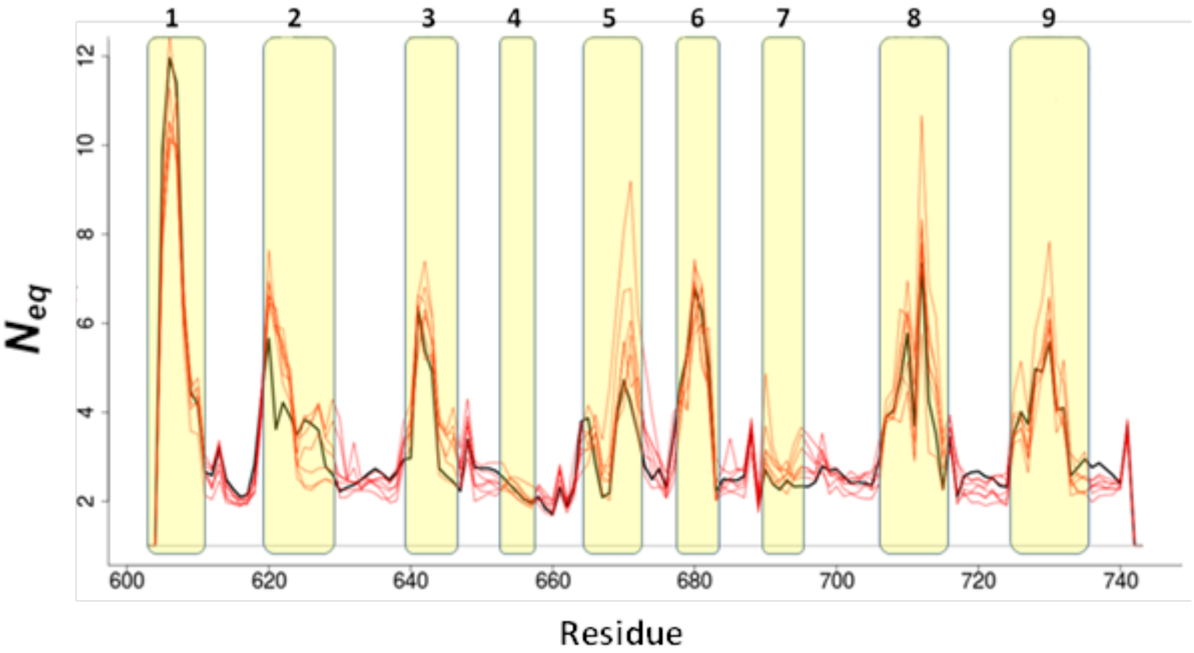

Figure S7. ***N_{eq}* values for all systems.** *N_{eq}* values of WT reference system are shown as black curve, while variant ones as red curves. Loops (numbering above) are delimited by yellow rectangles. Loop 2 is located at residues 620 to 622. Loop 10 is not reported here as it starts at residue 742.

Supplementary video 1. Position of the *Calf-1* domain within the integrin structure. Calf-1 domain is in yellow while the surrounding αIIb other domains are coloured in red and the β3 subunit in blue.

Supplementary video 2. Ribbon model of the *Calf-1* domain showing the location of the studied variant residues. β-strands are coloured in green and loops in yellow. Polymorphic residues are identified as red balls. N- and C- terminal ends are shown as yellow balls.
